# Neuron-specific gene NSG1 binds to and positively regulates sortilin ectodomain shedding *via* a metalloproteinase-dependent mechanism

**DOI:** 10.1016/j.jbc.2023.105446

**Published:** 2023-11-08

**Authors:** Malene Overby, Antonio Serrano-Rodriguez, Somayeh Dadras, Ann Kathrine Christiansen, Gözde Ozcelik, Stefan F. Lichtenthaler, Jason Porter Weick, Heidi Kaastrup Müller

**Affiliations:** 1 Translational Neuropsychiatry Unit, Department of Clinical Medicine, Aarhus University, Aarhus, Denmark; 2 Department of Neurosciences, University of New Mexico School of Medicine, Albuquerque, New Mexico, USA; 3 Department of Molecular Genetics and Microbiology, University of New Mexico School of Medicine, Albuquerque, New Mexico, USA; 4 German Center for Neurodegenerative Diseases (DZNE), Munich, Germany; 5 Neuroproteomics, School of Medicine, Klinikum Rechts der lsar, Technical University of Munich, Munich, Germany; 6 Munich Cluster for Systems Neurology (SyNergy), Munich, Germany

**Keywords:** sortilin receptor, ADAM10, Alzheimer’s disease, ectodomain shedding, NSG1, NSG2, progranulin, neurodegenerative disease, protein–protein interaction, yeast two-hybrid

## Abstract

Increasing evidence suggests that aberrant regulation of sortilin ectodomain shedding can contribute to amyloid-β pathology and frontotemporal dementia, although the mechanism by which this occurs has not been elucidated. Here, we probed for novel binding partners of sortilin using multiple and complementary approaches and identified two proteins of the neuron-specific gene (NSG) family, NSG1 and NSG2, that physically interact and colocalize with sortilin. We show both NSG1 and NSG2 induce subcellular redistribution of sortilin to NSG1- and NSG2-enriched compartments. However, using cell surface biotinylation, we found only NSG1 reduced sortilin cell surface expression, which caused significant reductions in uptake of progranulin, a molecular determinant for frontotemporal dementia. In contrast, we demonstrate NSG2 has no effect on sortilin cell surface abundance or progranulin uptake, suggesting specificity for NSG1 in the regulation of sortilin cell surface expression. Using metalloproteinase inhibitors and A disintegrin and metalloproteinase 10 KO cells, we further show that NSG1-dependent reduction of cell surface sortilin occurred *via* proteolytic processing by A disintegrin and metalloproteinase 10 with a concomitant increase in shedding of sortilin ectodomain to the extracellular space. This represents a novel regulatory mechanism for sortilin ectodomain shedding that is regulated in a neuron-specific manner. Furthermore, this finding has implications for the development of strategies for brain-specific regulation of sortilin and possibly sortilin-driven pathologies.

Sortilin (encoded by the *SORT1* gene) is a member of the vacuolar protein sorting ten protein (Vps10p) family of sorting receptors (1, 2) and is implicated in multiple forms of neurodegeneration. For example, sortilin is genetically associated with Alzheimer’s disease (AD) (3, 4), regulates several aspects of amyloid precursor protein (APP) and amyloid-β (Aβ) trafficking and processing (5, 6, 7, 8, 9), and facilitates endocytic uptake of apolipoprotein E (9), the most important genetic risk factor for late-onset AD (10). Sortilin has therefore been highlighted as a potential target to inhibit accumulation of APP-derived Aβ peptides, which cause the characteristic extracellular amyloid plaques seen in brains of AD patients. Sortilin is also a genetic risk factor for frontotemporal dementia (FTD) (11), and a sorting receptor for progranulin (PGRN) (12, 13, 14), a major causal gene for inherited FTD (15, 16), thus emphasizing a central role for sortilin in the molecular mechanisms underlying conditions associated with dementia (17, 18). Accordingly, a human monoclonal antibody directed against sortilin is currently in clinical trials (19, 20, 21) to block sortilin-mediated PGRN clearance in subjects at risk for or with FTD due to PGRN deficiency caused by heterozygous mutations in the *GRN* gene (15, 16).

Molecularly, sortilin contains a large Vps10p domain at the N terminus, a single-pass transmembrane domain, and a short intracellular C-terminal tail. Sortilin is subject to proteolytic cleavage by metalloproteinases in the extracellular juxtamembrane region (22, 23, 24). This process is known as ectodomain shedding (25) and leads to separation of the Vps10p ligand binding domain from the transmembrane domain and the intracellular C-terminal tail. Together, the transmembrane domain and C terminus form a Sortilin C-terminal *fra*gment that was recently found to create a unique form of plaque, termed “Sorfra” plaques, that deposit near amyloid plaques in patients with AD (26) and increase with AD progression (26, 27). Similarly, elevated levels of a truncated soluble form of sortilin that is equivalent to the ectodomain have been reported in brains from FTD cases with TDP-43 pathology (28). This results from an alternatively spliced *SORT1* mRNA transcript due to nuclear depletion of TDP-43 (28, 29). Like ectodomain shedding (22), alternative splicing of *SORT1* can affect synaptic plasticity through regulation of brain-derived neurotrophic factor sorting (30), thus supporting a link between unfavorable generation of a soluble sortilin receptor and mechanisms involved in neurodegenerative diseases.

Although proteolytic processing of sortilin has been known for decades, the molecular mechanisms of this processing, especially with regard to cell type–specific regulation, remain largely unexplored. Here, we identify neuron-specific protein family member 1 (NSG1) and NSG2 as novel interacting partners of sortilin. As their name indicates, both NSG1 and NSG2 are specifically expressed in neurons, and our results establish NSG1 as the first sortilin-interacting protein with a functional role in the regulation of sortilin ectodomain shedding. We demonstrate that NSG1-induced sortilin ectodomain shedding is dependent on A disintegrin and metalloproteinase 10 (ADAM10) activity. The NSG1-induced increase in sortilin ectodomain shedding results in a decrease in sortilin cell surface expression, which leads to a concomitant decrease in PGRN uptake. Together, these findings uncover novel interactions between NSG1, NSG2, and sortilin and identify a new regulatory mechanism for sortilin ectodomain shedding that is unique to neurons.

## Results

### NSG1 and NSG2 are novel sortilin-binding proteins

**Figure 1:**
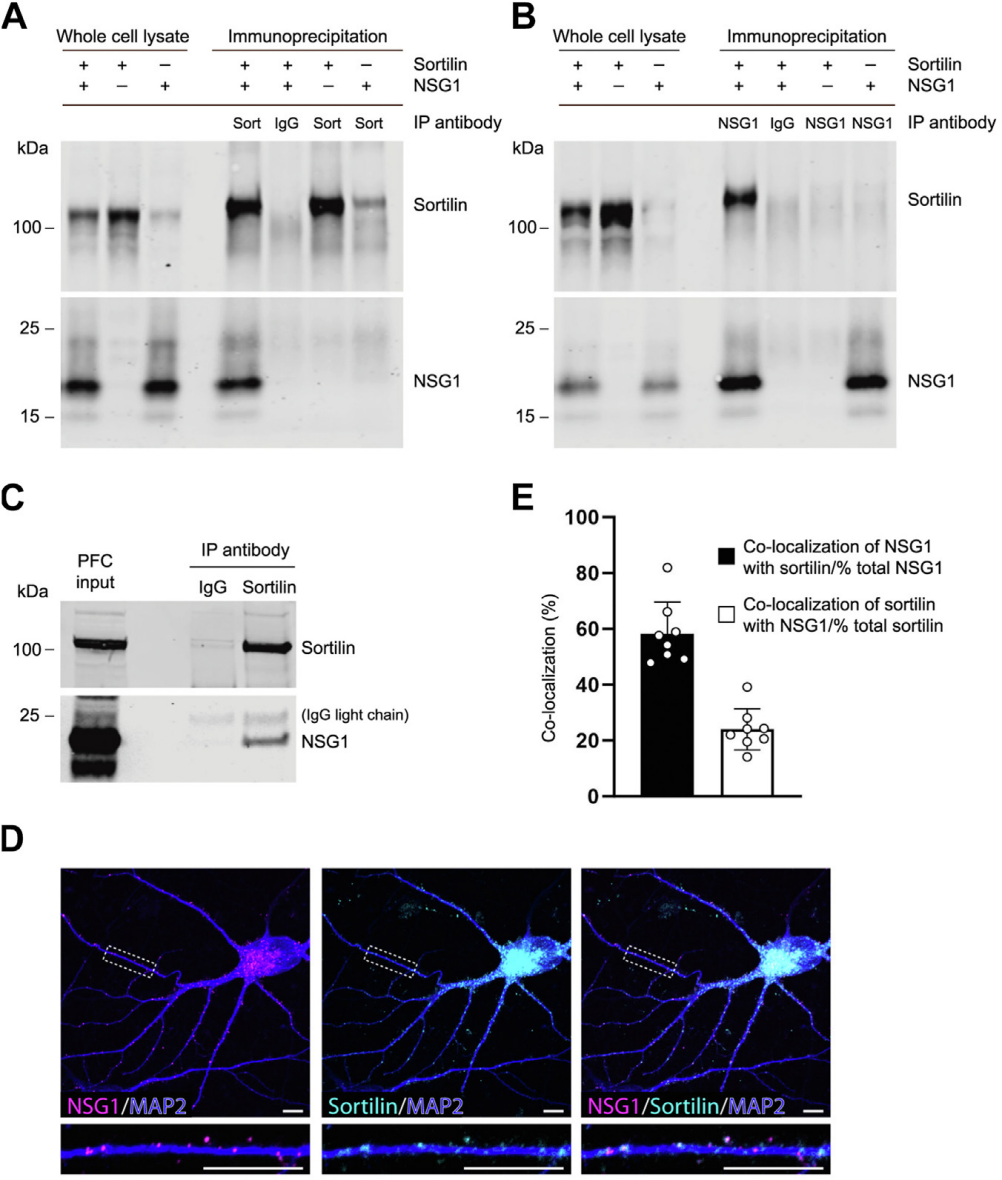
**Confirmation of NSG1 as a sortilin-interacting partner.***A*, NSG1 coimmunoprecipitates with sortilin in transfected HEK293MSR cells. HEK293MSR cells transfected with sortilin and NSG1 together or alone were subjected to control IgG or anti-sortilin (SORT) antibody immunoprecipitation (IP). Western blots of the immunoprecipitates and whole-cell lysates were probed with the antibodies indicated on the *right* of the blot. *B*, reverse coimmunoprecipitation. Sortilin coimmunoprecipitates with NSG1 in transfected HEK293MSR cells. HEK293MSR cells transfected with sortilin and NSG1 together or alone were subjected to control IgG or anti-NSG1 antibody immunoprecipitation. Western blots of the immunoprecipitates and whole-cell lysates were probed with the antibodies indicated on the *right* of the blot. *C*, endogenous sortilin–NSG1 interaction. Rat (10 weeks) prefrontal cortex (PFC) lysates were immunoprecipitated with control IgG or anti-sortilin antibody. Western blots of the immunoprecipitates and total prefrontal cortex lysate were probed with the antibodies indicated on the *right* of the blot. *D*, colocalization of endogenous sortilin and NSG1 in cultured hippocampal neurons (DIV17). Cultured hippocampal neurons were immunostained with anti-sortilin, anti-NSG1, and anti-MAP2 antibodies. Images were acquired with confocal microscopy; the scale bar represents 10 μm. *E*, quantification of colocalization of sortilin with NSG1. Bars represent mean ± SD for n = 7 neurons assayed for each group. DIV, days *in vitro*; HEK, human embryonic kidney; IgG, immunoglobulin G; NSG, neuron-specific protein family member.

To identify proteins interacting with the cytosolic C-terminal domain of sortilin, we used residues 779 to 831 of sortilin as bait in a yeast two-hybrid screen against a human brain complementary DNA (cDNA) library (Fig. S1). From approximately 4 × 10^6^ yeast transformants, a total of 63 different putative sortilin interactors were recovered (Table S1). Because NSG1 was previously reported to be involved in trafficking of neurotensin receptors 1 to 2 (31), alpha-amino-3-hydroxy-5-methyl-4-isoxazolepropionate (AMPA) receptor subunits (32, 33, 34) and in the processing of APP (35), we decided to investigate the nature of sortilin–NSG1 interactions further. First, we confirmed that NSG1 was a positive candidate for sortilin interaction by retransforming NSG1 into yeast expressing either the sortilin bait construct or empty vector. Activation of reporter genes was observed only in the presence of sortilin. Using coimmunoprecipitation, the anti-sortilin antibody but not control antibody immunoglobulin G (IgG), coimmunoprecipitated a complex between NSG1 and sortilin in detergent extracts from transiently transfected human embryonic kidney (HEK) 293MSR cells (Fig. 1*A*). The reverse was also true, with the anti-NSG1 antibody able to pull down sortilin from HEK293MSR cells transfected with both constructs (Fig. 1*B*). Furthermore, we confirmed an endogenous interaction *in vivo via* immunoprecipitation of a sortilin–NSG1 complex in lysates from rat prefrontal cortex (Fig. 1*C*). These experiments provide additional biochemical evidence of a physical interaction between sortilin and NSG1.

We further examined the spatial localization of sortilin and NSG1 in cultured hippocampal neurons after 17 days *in vitro*. Total protein expression was analyzed in Triton-permeabilized cells with antibodies directed towards the intracellular C-terminal region of sortilin and the intracellular N-terminal region of NSG1 (see methods). Fig. 1*D* illustrates a representative neuron that demonstrates extensive colocalization in peri-nuclear soma, as well as throughout MAP2^+^ dendritic arbors. Peri-nuclear staining likely represents Golgi localization, where both proteins show robust expression (36, 37, 38). Semiautomated colocalization analysis of dendritic puncta revealed that 24% of sortilin puncta colocalized with NSG1, while a remarkable 58% of NSG1 puncta colocalized with sortilin (Fig. 1*E*, n = 7). Correlation analysis of dendritic puncta revealed a Pearson’s correlation coefficient of 0.52 ± 0.03 (n = 7), and Manders’ coefficients of 0.67 ± 0.03 (M1; sortilin/NSG1) and 0.26 ± 0.04 (M2; NSG1/sortilin). Together, these data suggest that at least a subset of NSG1 and sortilin traffic together in intracellular vesicles (37, 38, 39, 40).

**Figure 2:**
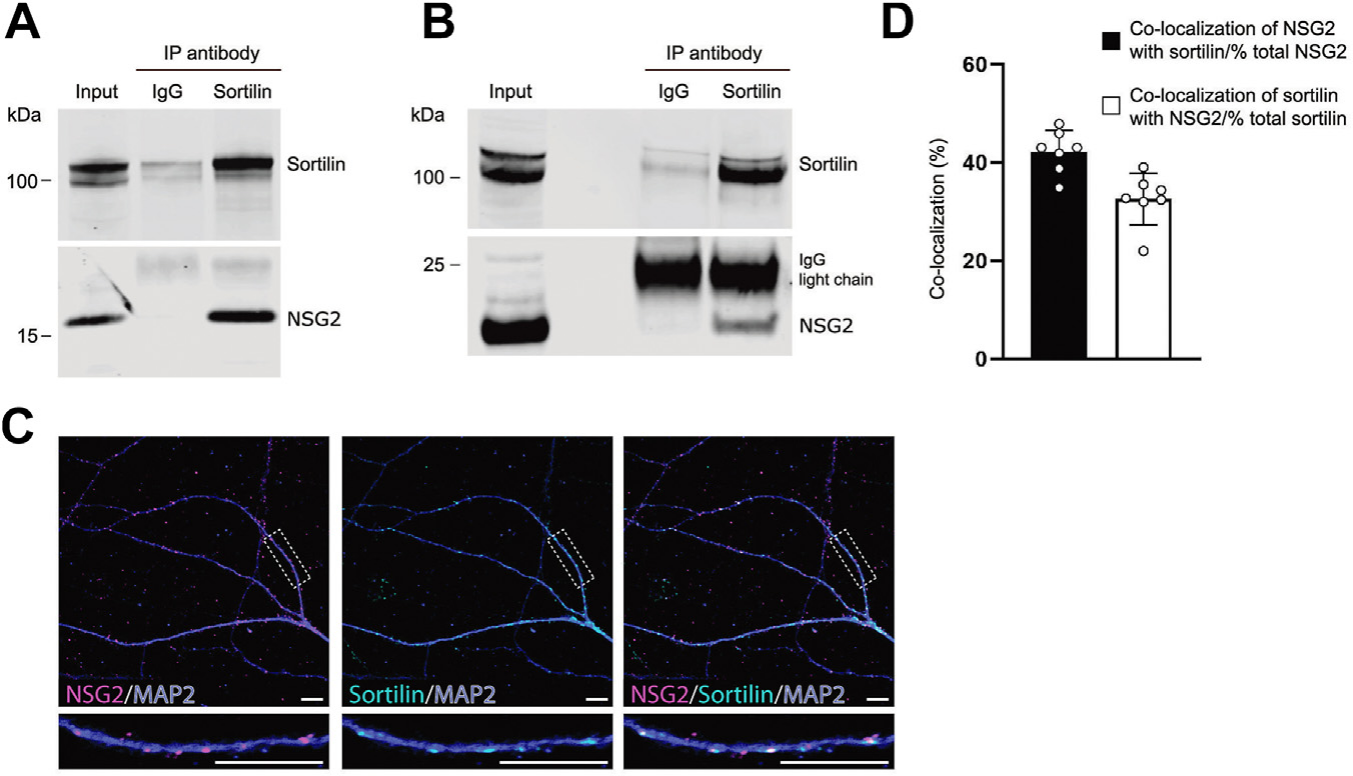
**Sortilin binds to and colocalizes with NSG2.***A*, NSG2 coimmunoprecipitates with sortilin in transfected HEK293MSR cells. HEK293MSR cells transfected with sortilin and NSG2 were subjected to control IgG or anti-sortilin antibody immunoprecipitation (IP). Western blots of the immunoprecipitates and whole-cell lysates were probed with the antibodies indicated on the *right* of the blot. *B*, endogenous sortilin–NSG2 interaction. Rat (10 weeks) prefrontal cortex lysates were immunoprecipitated with control IgG or anti-sortilin antibody. Western blots of the immunoprecipitates and total prefrontal cortex lysate were probed with the antibodies indicated on the *right* of the blot. *C*, colocalization of endogenous sortilin and NSG2 in cultured hippocampal neurons (DIV17). Cultured hippocampal neurons were immunostained with anti-sortilin, anti-NSG2, and anti-MAP2 antibodies. Images were acquired with confocal microscopy; the scale bar represents 10 μm. *D*, quantification of colocalization of sortilin with NSG1. Bars represent mean ± SD for n = 8 neurons assayed for each group. DIV, days *in vitro*; HEK, human embryonic kidney; IgG, immunoglobulin G; NSG, neuron-specific protein family member.

NSG1 shows extensive colocalization with its family member NSG2 (39). We therefore tested whether sortilin also binds to NSG2. We found that NSG2 coimmunoprecipitated with sortilin in transfected HEK293MSR cells (Fig. 2*A*) and in rat prefrontal cortex (Fig. 2*B*). In cultured hippocampal neurons, sortilin also colocalized with NSG2 (Fig. 2*C*), and to a slightly higher extent than NSG1 (32.6 ± 5.2% *versus* 24 ± 7.4%; *p* = 0.024, unpaired *t* test, n = 8) (Figs. 1*E* and 2*D*). This was despite the fact that a considerably smaller fraction of total NSG2 puncta colocalized with sortilin (42.2 ± 4.3% *versus* 58.3 ± 11.3%; *p* = 0.004, unpaired *t* test, n = 8) (Figs. 1*E* and 2*D*). Correlation analysis found similar Pearson’s (0.48 ± 0.05) and Manders’ M1 (NSG2/sortilin; 0.29 ± 0.06) but smaller M2 (sortilin/NSG2; 0.38 ± 0.04) for NSG2. Overall, NSG1 and NSG2 colocalize with sortilin in the peri-nuclear Golgi apparatus, as well as in discrete complexes along neuronal dendrites.

### Redistribution of sortilin to NSG1/NSG2 enriched compartments

**Figure 3:**
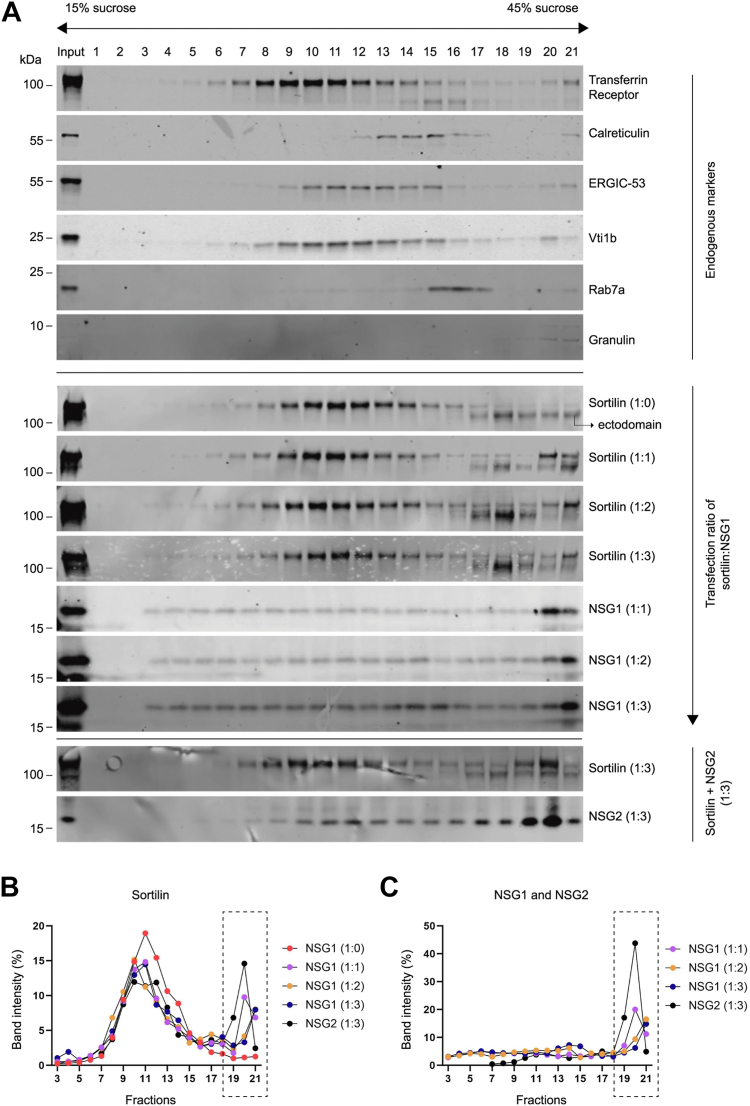
**Subcellular localization of sortilin determined by sucrose density gradient fractionation.***A*, HEK293MSR cells were cotransfected with sortilin and pcDNA3, sortilin, and NSG1 (at DNA ratios of 1:1, 1:2, and 1:3) or sortilin and NSG2 in a 1:3 DNA ratio. Cells were lysed and fractionated by velocity sedimentation through a discontinuous sucrose gradient. Western blots of equal volume aliquots of fractions were probed with the antibodies indicated on the *right* of the blots. *B*, signal intensities of full-length sortilin were quantified and the relative distribution in each of the sucrose density fractions is plotted as percentage of total sortilin intensity. *C*, signal intensities of NSG1 and NSG2 were quantified and the relative distribution in each of the sucrose density fractions is plotted as percentage of total protein intensity. HEK, human embryonic kidney; NSG, neuron-specific protein family member.

Previous studies have revealed a role for NSG1 in endosomal trafficking and sorting of membrane proteins (41). Following our data showing that NSG1 binds to sortilin, we asked if this interaction has functional effects on sortilin localization. First, we employed subcellular fractionation *via* sucrose density gradient centrifugation to investigate the distribution of sortilin in HEK293MSR cells when coexpressed with increasing amounts of NSG1. Antibodies directed against specific markers were used for identification of subcellular compartments across the isolated cellular fractions (Fig. 3*A*). Sortilin localization showed considerable overlap in distribution with the transferrin receptor (TfR), which is localized on the plasma membrane and in early endosomal compartments (42). Overlap with the Golgi protein ERGIC-53 (43) and with Vti1b, which localizes to the *trans-*Golgi network and to the late endosomal compartment (44) was also observed (Fig. 3*A*). In the presence of either NSG1 or NSG2, a significant redistribution of full-length sortilin from fractions 10 to 14 toward the high-density fractions 17 to 21 was detected (Fig. 3, *A* and *B*). Importantly, when sortilin is transfected with NSG1 in a 1:1 ratio, the relocalized sortilin appears strongest in fraction 20, which under these conditions is also the fraction that contains the most NSG1 (Fig. 3, *A*–*C*). In contrast, when sortilin is transfected with NSG1 in a 1:2 and 1:3 ratio, sortilin shows up strongest in fraction 21, which under these conditions is also the fraction that contains the largest amounts of NSG1. These findings indicate that a subset of sortilin traffics specifically with NSG1 in a dose-dependent manner (Fig. S2) and relocate to high-density fractions positive for lysosomal granulin (Fig. 3*A*) (45). Similarly, when sortilin is transfected with NSG2 (in a 1:3 ratio), sortilin shows up strongest in the fraction with most NSG2 (fraction 20, Fig. 3, *A*–*C*). Interestingly, both NSG1 and NSG2 displayed unique localization patterns with particularly strong enrichment in fractions 20 to 21 (Fig. 3, *A* and *C*). It is also noteworthy how the proteolytically cleaved sortilin ectodomain localizes exclusively to the high-density fractions with a sharp increase in intensity in fraction 18 when sortilin is transfected with NSG1 in a 1:2 and 1:3 ratio, but not when transfected with NSG2 (Fig. S3).

### NSG1 regulates sortilin-mediated PGRN uptake by reducing sortilin cell surface expression

**Figure 4:**
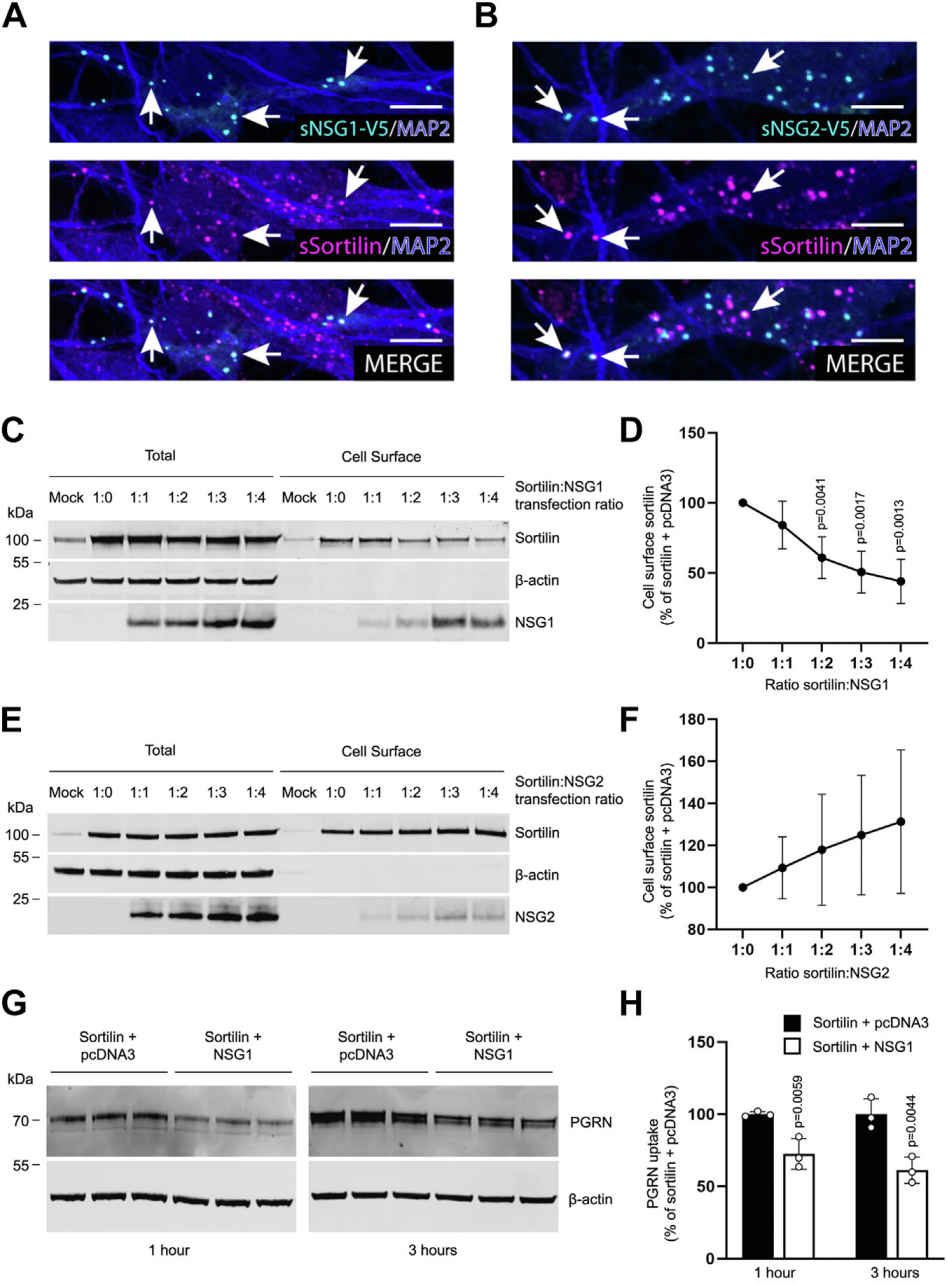
**NSG1 reduces sortilin cell surface expression and PGRN uptake.***A*, representative confocal images showing plasma membrane colocalization of sortilin and NSG1 in cultured hippocampal neurons (DIV14) immunostained with anti-sortilin, anti-V5, and anti-MAP2 antibodies; the scale bar represents 10 μm. *B*, representative confocal images showing plasma membrane colocalization of sortilin and NSG2 in cultured hippocampal neurons (DIV14) immunostained with anti-sortilin, anti-V5, and anti-MAP2 antibodies; the scale bar represents10 μm. *C*, representative Western blot of whole-cell lysate and biotin-labeled fractions from HEK293MSR cells transfected with sortilin and NSG1 at DNA ratios of 1:0, 1:1, 1:2, 1:3, and 1:4. Primary antibodies are indicated on the *right* of the blot. *D*, graph showing the proportion of cell surface sortilin, normalized to total sortilin, and expressed as percentage of control (sortilin + pcDNA3). Quantified results are from five independent experiments; one sample *t* test compared to control. *E*, representative Western blot of whole-cell lysate and biotin-labeled fractions from HEK293MSR cells transfected with sortilin and NSG2 at DNA ratios of 1:0, 1:1, 1:2, 1:3, and 1:4. Primary antibodies are indicated on the *right* of the blot. *F*, graph showing the proportion of cell surface sortilin, normalized to total sortilin, and expressed as percentage of control (sortilin + pcDNA3). Quantified results are from four independent experiments. *G*, HEK293MSR cells transfected with sortilin and pcDNA3 or sortilin and NSG1 at DNA ratios of 1:3 were incubated with HA-PGRN conditioned media for 1 or 3 h. Internalized HA-PGRN was recovered from whole-cell lysates and analyzed by Western blotting using an anti-HA antibody. *H*, *bar graphs* showing β-actin normalized density of PGRN, expressed as percentage of control (sortilin + pcDNA3), and comparisons between groups were conducted using a one-tailed *t* test. The data are representative of two independent experiments. DIV, days *in vitro*; HA, hemagglutinin; HEK, human embryonic kidney; NSG, neuron-specific protein family member; PGRN, progranulin.

Sortilin is a cell surface receptor for a number of ligands (46), and NSGs have recently been found on the plasma membrane of neurons (39). Thus, we next determined if NSG1 and NSG2 colocalize with sortilin at the cell surface of cultured hippocampal neurons (14 days *in vitro*) expressing full-length NSG1 or NSG2 with a V5 tag located on their extracellular C termini. Nonpermeabilized cells were incubated with anti-V5 antibody as well as anti-sortilin antibody directed towards the extracellular N-terminal region of sortilin. Subsequent permeabilization and staining for MAP2 allowed us to visualize surface NSG1/sortilin complexes along dendritic regions. Figure 4 illustrates representative confocal images that demonstrate 51.6 ± 4.4% of sortilin punctae colocalized with NSG1 (Fig. 4*A*) while 56.5 ± 5.8% colocalized with NSG2 (Fig. 4*B*) on the plasma membrane.

Given that sortilin interacts with NSG1 and NSG2 at the cell surface, we asked if the interaction has functional effects on sortilin cell surface abundance. Using cell surface biotinylation, we found that NSG1 altered the cell surface expression of sortilin in a dose-dependent manner (Fig. 4, *C* and *D*), where increasing amounts of NSG1 caused significant decreases in cell surface sortilin. In contrast, NSG2 did not change sortilin cell surface expression (Fig. 4, *E* and *F*). Total sortilin protein levels were not affected by the increase in levels of NSG1 or NSG2 (Fig. 4, *C* and *E*). Increased expression of NSG1 and NSG2 also led to an increase in the amount of NSG1 and NSG2 present at the cell surface (Fig. 4, *C* and *E*).

To assess functional consequences of the NSG1-induced reduction in sortilin cell surface levels, we investigated sortilin-mediated PGRN uptake from conditioned media. Cells cotransfected with NSG1 exhibited reduced uptake of PGRN, as measured by accumulation of hemagglutinin (HA)-tagged PGRN in the cell lysate, consistent with reduced sortilin available at the cell surface for PGRN binding and uptake (Fig. 4, *G* and *H*). In contrast, NSG2 had no functional effect on sortilin-mediated PGRN uptake (Fig. S4).

### NSG1 reduces sortilin cell surface expression through a trafficking-independent mechanism

**Figure 5:**
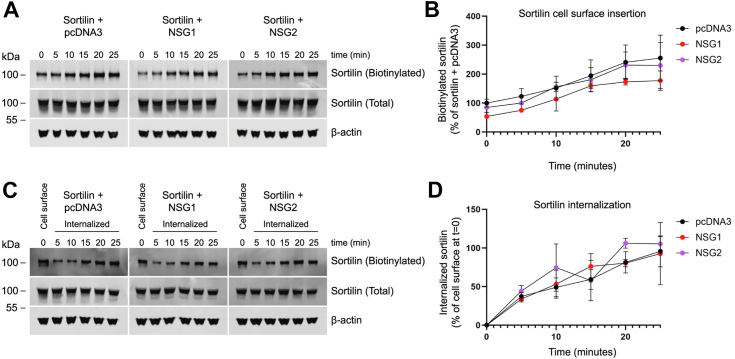
**NSG1 does not change intracellular trafficking rates of sortilin.***A*, representative Western blot of cell surface insertion assay. HEK293MSR cells transfected with sortilin together with pcDNA3, NSG1, or NSG2 at DNA ratios of 1:3 were labeled with biotin and transferred to 37 °C for the indicated times to allow for receptor trafficking. Pools of accumulated cell surface biotinylated sortilin were recovered with streptavidin beads. Primary antibodies are indicated on the *right* of the blot. *B*, graph showing the proportion of cell surface sortilin, normalized to total sortilin, and expressed as percentage of control (sortilin + pcDNA3). Quantified results are from three independent experiments. *C*, representative Western blot of internalization assay. HEK293MSR cells transfected with sortilin together with pcDNA3, NSG1, or NSG2 at DNA ratios of 1:3 were labeled with biotin. One set of cells was lysed to examine total surface biotin-labeled sortilin at *t* = 0, while the rest were incubated at 37 °C to allow for receptor internalization and at the indicated times stripped of remaining surface biotin with MESNa. Intracellular pools of biotinylated sortilin were recovered with streptavidin beads. Primary antibodies are indicated on the *right* of the blot. *D*, graph showing the proportion of internalized sortilin, normalized to total input sortilin, and expressed as percentage of total surface-biotinylated sortilin at *t* = 0. Quantified results are from three independent experiments. HEK, human embryonic kidney; NSG, neuron-specific protein family member.

To determine whether the NSG1-mediated decrease in sortilin cell surface expression is caused by trafficking events, we performed cell surface biotinylation assays under trafficking-permissive conditions. The insertion rate of sortilin in the surface membrane (5.8%/min ± 0.43%) was not changed by coexpression with NSG1 (5.7%/min ± 0.48%) or NSG2 (5.9%/min ± 0.76%) (Fig. 5, *A* and *B*). To measure sortilin internalization, receptors residing on the cell surface were first labeled with cleavable biotin and then incubated at 37 °C for variable amounts of times to allow endocytosis. Biotin was stripped from proteins that remained at the cell surface to select for recovery of internalized proteins only. The internalization rate of sortilin in the presence of NSG1 (5.0%/min ± 0.54%) or NSG2 (5.9%/min ± 0.97%) was however not different from the average rate of internalization measured under control conditions (4.8%/min ± 0.68%) (Fig. 5, *C* and *D*). Collectively, these data suggest that the NSG1-induced decrease in sortilin cell surface expression and reduction in PGRN uptake cannot be attributed to an increase in sortilin internalization, or to a reduction in the replenishment of new receptors to the cell surface.

### NSG1 increases ectodomain shedding of sortilin

**Figure 6:**
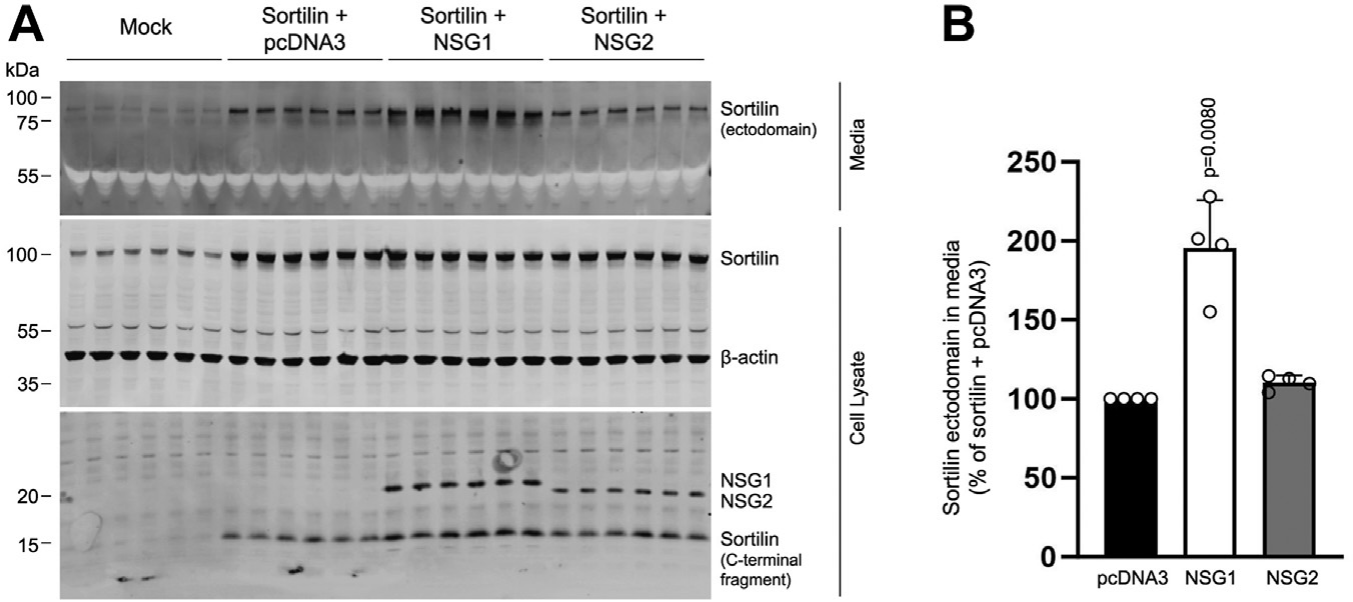
**NSG1 increases ectodomain shedding of sortilin.***A*, representative Western blot of conditioned media and whole-cell lysates from mock-transfected HEK293MSR cells and HEK293MSR cells transfected with sortilin together with pcDNA3, NSG1, or NSG2 at DNA ratios of 1:3. Primary antibodies are indicated on the right of the blots. *B*, *bar graph* showing the relative abundance of sortilin ectodomain in the media normalized to total sortilin and expressed as percentage of control (sortilin + pcDNA3). Quantified results are from four independent experiments; one sample *t* test compared to control. HEK, human embryonic kidney; NSG, neuron-specific protein family member.

Sortilin is subject to ectodomain shedding (22, 23, 24); the process by which the extracellular ligand–binding domain of sortilin is separated from the membrane bound intracellular C-terminal tail through juxtamembrane cleavage by metalloproteinases. Ectodomain shedding is emerging as an important cellular mechanism to control the abundance and activity of cell surface receptor levels (25, 47). To test whether NSG1 and NSG2 affect sortilin ectodomain shedding, we used Western blotting analysis to examine levels of accumulated sortilin ectodomain in conditioned media from HEK293MSR cells. Specific recognition of the sortilin ectodomain was confirmed using antibodies directed against the N- and C-terminal domains of sortilin, respectively. While both antibodies detected full-length sortilin in total cell lysate, the sortilin ectodomain was only recognized by the antibody directed against an epitope in the N-terminal domain (Fig. S5). Consistent with previous studies (23), low levels of endogenous sortilin ectodomain could be detected in media collected from mock-transfected cells (Fig. 6*A*, lanes 1–6), indicating that the process of ectodomain shedding is not related to the overexpression of sortilin. Compared to untransfected controls, increased levels of soluble sortilin ectodomain were detected in media from cells transfected with sortilin and control pcDNA3.1 empty vector (Fig. 6*A*, lanes 7–12). Critically, when combined with NSG1 overexpression, we found significantly increased levels of secreted sortilin ectodomain compared with cells transfected with sortilin and empty vector (Fig. 6, *A* and *B*, lanes 13–18). In contrast, the presence of NSG2 did not change sortilin ectodomain in the media (Fig. 6, *A* and *B*, lanes 18–24). Taken together, our data suggest that the NSG1-induced decrease in sortilin cell surface abundance is caused by increased ectodomain shedding of sortilin.

### NSG1 induces sortilin ectodomain shedding through an ADAM10-dependent mechanism

**Figure 7:**
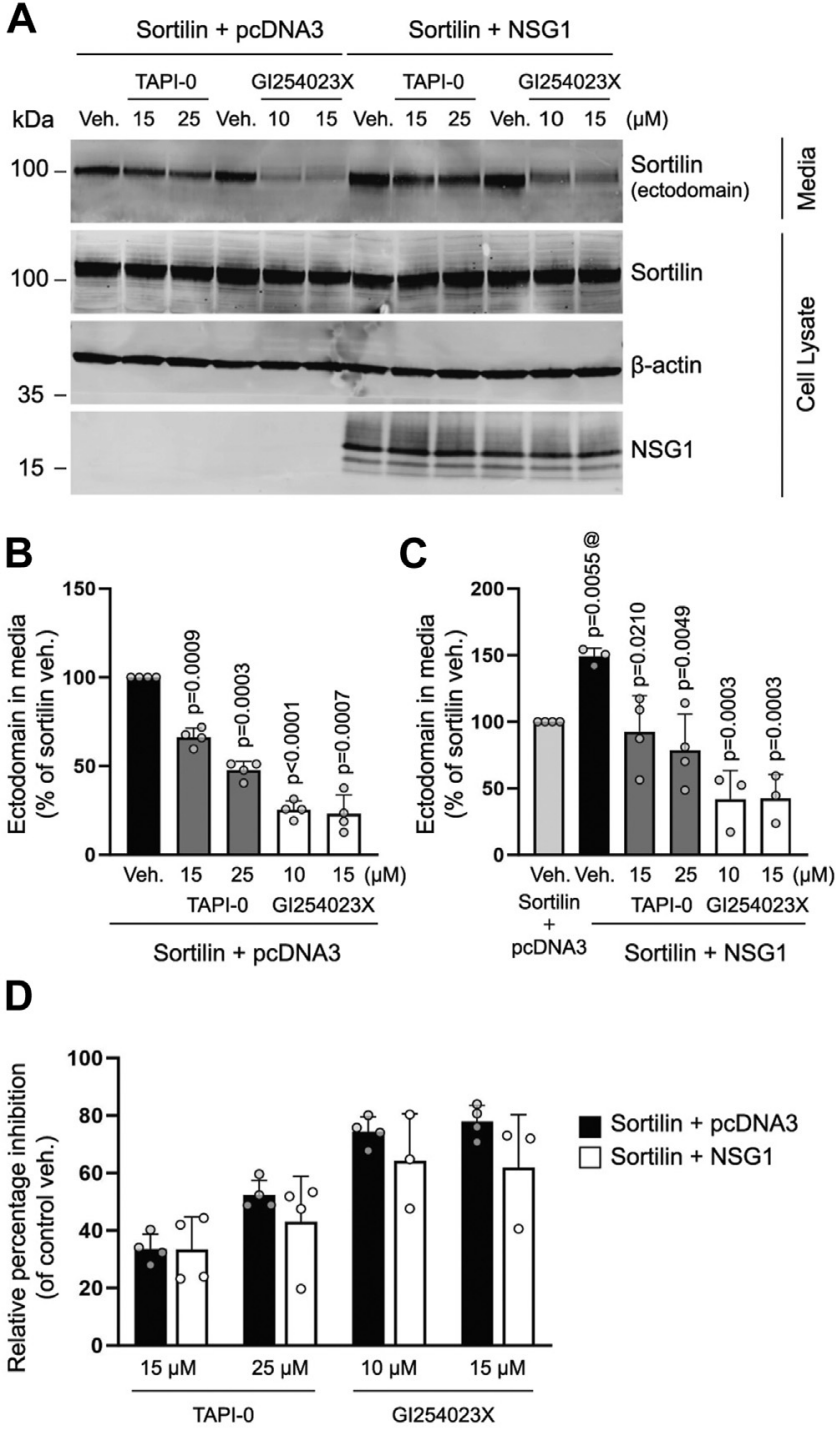
**Metalloproteinase inhibitors prevent NSG1-induced sortilin ectodomain shedding.***A*, representative Western blot of conditioned media and whole-cell lysates from HEK293MSR cells transfected with sortilin and pcDNA3 or sortilin and NSG1 at DNA ratios of 1:3 treated with vehicle (veh.), the ADAM17 inhibitor TAPI-0, or the ADAM10 inhibitor GI254023X as indicated. Primary antibodies are indicated on the *right* of the blots. *B*, *bar graph* showing the relative abundance of sortilin ectodomain in the media normalized to total sortilin and expressed as percentage of veh. Quantified results are from four independent experiments, one-sample *t* test. *C*, *bar graph* showing the relative abundance of sortilin ectodomain in the media normalized to total sortilin and expressed as percentage of control (sortilin + pcDNA3 veh.). @ compared to sortilin + pcDNA3 veh., one-sample *t* test; others, compared to sortilin+NSG1 veh., one-way ANOVA with Dunnett’s multiple comparisons test (F_(4, 12)_ = 11.54, *p* = 0.0004). Quantified results are from 3 to 4 independent experiments. *D*, *bar graphs* showing the relative percentage shedding inhibition in NSG1-expressing conditions compared to control conditions (pcDNA3). ADAM, A disintegrin and metalloproteinase; HEK, human embryonic kidney; NSG, neuron-specific protein family member.

**Figure 8:**
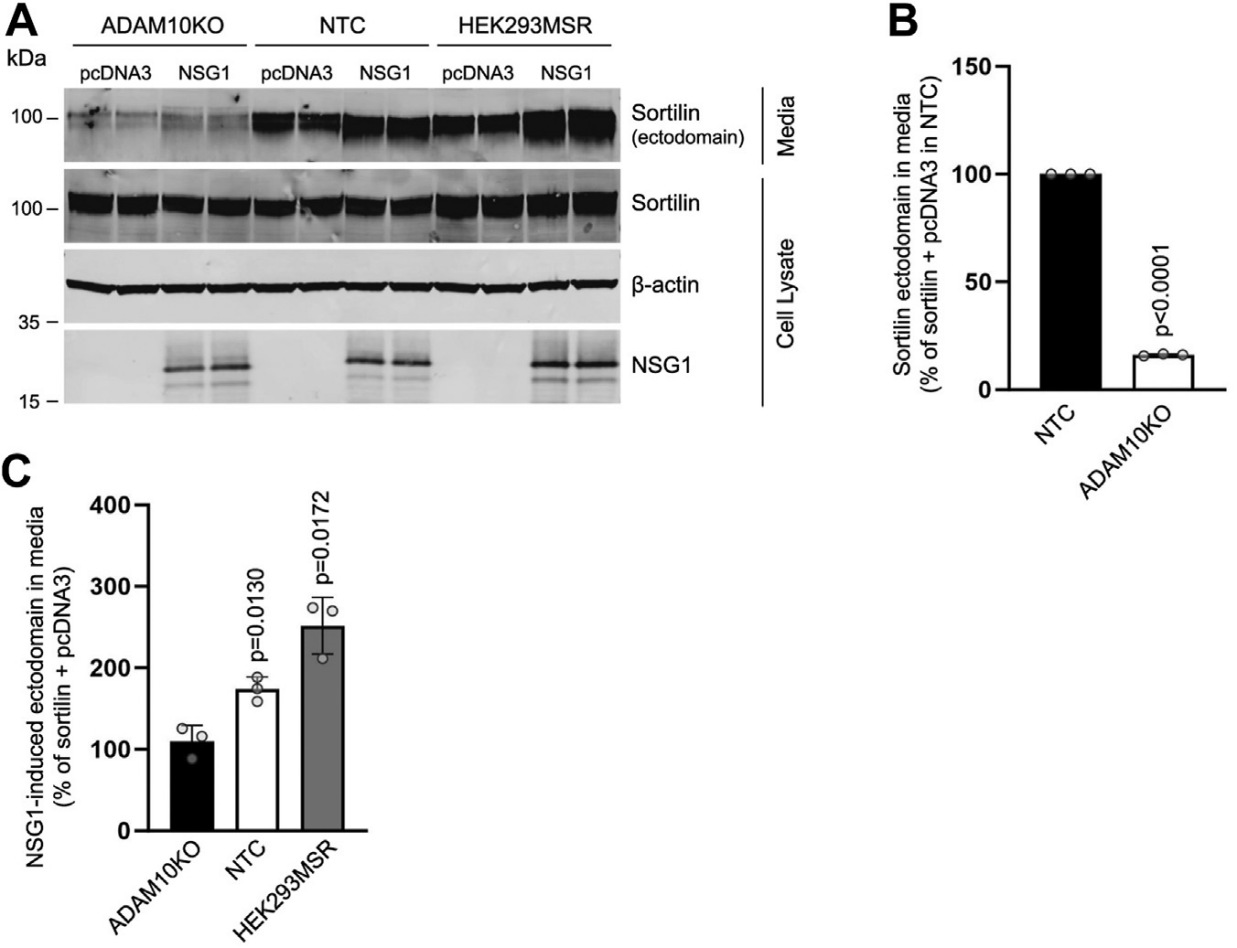
**ADAM10 is required for NSG1 to stimulate sortilin cleavage.***A*, representative Western blot of conditioned media and whole-cell lysates from ADAM10 KO HEK293T cells, nontargeting control (NTC) HEK293T cells, and HEK293MSR cells transfected with sortilin and pcDNA3 or sortilin and NSG1 at DNA ratios of 1:3. Primary antibodies are indicated on the *right* of the blots. *B*, *bar graph* showing sortilin ectodomain in the media normalized to total sortilin for sortilin + pcDNA3 transfected ADAM10KO cells expressed as percentage of sortilin + pcDNA3 transfected NTC cells. Quantified results are from three independent experiments, one-sample *t* test. *C*, *bar graph* showing NSG1-induced sortilin ectodomain in the media normalized to total sortilin and expressed as percentage of sortilin + pcDNA3 for each cell type. Quantified results are from three independent experiments, one-sample *t* test. ADAM, A disintegrin and metalloproteinase; HEK, human embryonic kidney; NSG, neuron-specific protein family member.

ADAM10 and the related protein ADAM17 are the major enzymes responsible for ectodomain shedding of many membrane proteins (25, 48). A previous study demonstrated that ADAM10 is the primary enzyme responsible for sortilin ectodomain shedding (22). To test the involvement of ADAM10 and ADAM17 in NSG1-induced sortilin ectodomain shedding, we examined the ability of inhibitors of ADAM10 and ADAM17 to impede the release of sortilin ectodomain into the media. In the absence of NSG1, sortilin ectodomain shedding was significantly inhibited by both the ADAM17 inhibitor TAPI-0 and the ADAM10 inhibitor GI254023X (Fig. 7, *A* and *B*). However, inhibition of ADAM10 was more effective in blocking sortilin ectodomain shedding, consistent with the notion that ADAM10 is the primary enzyme involved in the shedding of sortilin (Fig. 7, *A* and *B*) (22). If ADAM10 and ADAM17 are involved in NSG1-induced sortilin ectodomain shedding, then inhibition of the respective enzymes should block the increase in sortilin ectodomain shedding induced by NSG1. The results show that both TAPI-0 and GI254023X abolished the effect of NSG1 on sortilin shedding (Fig. 7, *A* and *C*). To rule out the possibility that this effect was due to reductions in NSG1-independent shedding, we calculated the relative percentage shedding inhibition in NSG1-expressing conditions compared to control conditions. In the presence of NSG1, TAPI-0, and GI254023X inhibited the same relative fraction of sortilin ectodomain shedding as in the absence of NSG1 (Fig. 7*D*). Because GI254023X and TAPI-0 are selective but not specific, we also examined sortilin ectodomain shedding in HEK293T cells with CRISPR/Cas9-induced knockout of ADAM10 (ADAM10KO) and in nontargeting control (NTC) cells (Fig. S6). HEK293MSR cells were included as control. Ectodomain shedding of sortilin was greatly reduced in ADAM10KO cells compared with NTC cells (Fig. 8, *A* and *B*) and NSG1 was unable to stimulate sortilin cleavage in the absence of ADAM10 (Fig. 8, *A* and *C*). Taken together, these results indicate that ADAM10 is required for the mechanism by which NSG1 induces sortilin ectodomain shedding.

## Discussion

Sortilin is a multifunctional receptor, playing critical roles in protein trafficking and signaling. To understand how sortilin function is regulated by interacting proteins, we performed a yeast two-hybrid screen in search of novel binding partners of sortilin. Among the positive candidates, we focused our further efforts on NSG1, a protein previously shown to bind and regulate the trafficking of several disease-relevant proteins, including AMPA receptor subunits (32, 33, 34), L1/NgCAM (49), TrkB (50), and to be involved in amyloidogenic processing of APP (35). We initially confirmed interaction and colocalization of NSG1 with sortilin in mammalian cells transiently coexpressing both proteins, in rat brain, and in cultured hippocampal neurons. NSG1 belongs to the neuron-specific gene family (51), which also includes NSG2/P19 and NSG3/calcyon, a group of small, single-transmembrane spanning proteins that are highly enriched in neurons, where they localize to the plasma membrane, the *trans-*Golgi network and multiple endolysosomal compartments (39). We found that NSG2, which shares 52.4% overall amino acid sequence identity with NSG1, also binds and colocalizes with sortilin. In accordance with the literature, we show here that NSG1 and NSG2 are broadly distributed across most organelle membranes as shown by subcellular fractionation, although with considerable enrichment in high-density fractions positive for lysosomal granulin peptides (45). Sortilin was distributed across the fractionated samples in agreement with previous findings (52, 53), and its overall distribution was not altered by coexpression with NSG1 or NSG2. However, a subset of sortilin was found to redistribute and colocalize with NSG1 and NSG2 in the high-density fractions. This finding indicates that NSG1 and NSG2 *via* their binding to sortilin can relocate sortilin to a specific compartment, which under control conditions (in the absence of NSG1 or NSG2) is largely devoid of sortilin. It would be interesting to further explore the nature of these fractions, especially considering recent results showing increased intraneuronal sortilin aggregation in a specific type of lysosomal structure, termed granulovacuolar degeneration bodies, which is associated with AD pathology (54).

In our experiments, the proportion of endogenous surface sortilin in HEK293MSR cells is approximately 10% of total cellular sortilin, which is similar to estimates from other cell types (38, 55), indicating that mechanisms regulating sortilin cell surface expression may be common across different cell types. When overexpressing sortilin, we find that this distribution is increased to 15%. Here, we demonstrate that NSG1 dose-dependently reduced the steady-state level of sortilin molecules at the cell surface by more than 50%. A role for NSG1 in receptor sorting and recycling to the cell surface has previously been demonstrated for the AMPA receptor subunit GluR2 (32), TfR (33), and neurotensin receptor 1 to 2 (31). However, we found that trafficking rates for sortilin membrane insertion and internalization were unaffected by coexpression with NSG1. Instead, we found that NSG1 downregulates sortilin cell surface expression by inducing sortilin ectodomain shedding. In contrast, NSG2 had no effect on these parameters, thus highlighting the specificity of NSG1 in regulating sortilin ectodomain shedding. With these findings, NSG1 is the first sortilin-interacting protein identified to act on this level of posttranslational regulation. While NSG1 is best known for its role in endosomal trafficking, previous work indicates that NSG1 can regulate Aβ levels by interfering with APP proteolytic processing (35). However, it remains unclear whether the effect of NSG1 on APP processing occurs in intracellular compartments or at the plasma membrane (35, 41). Interestingly, NSG3, the third member of the NSG family, has been shown to stimulate ectodomain shedding of neuregulin 1 *via* an endocytosis-dependent mechanism (56). NSG3, which shares 32.9% amino acid sequence identity with NSG1, has a predicted membrane topology opposite to that of NSG1 and NSG2. It will be interesting to test if NSG3 also binds to and regulates proteolytic processing of sortilin. Regardless, our finding that NSG1 can induce sortilin ectodomain shedding adds support to a previously unrecognized function of NSG proteins in regulating ectodomain shedding of membrane proteins specifically in neurons.

Although many studies have addressed the function of sortilin in terms of expression and sorting, little is known about the mechanism of its ectodomain shedding. Sortilin ectodomain shedding has been demonstrated in different cell types, including neurons, microglia, and immortalized cell lines (22, 23, 57). The cell surface is considered the main cellular platform for ectodomain shedding of sortilin (22, 23, 24) and other membrane proteins (25) but sortilin ectodomain shedding has also been identified in intracellular compartments (22). In our experiments, we measured sortilin ectodomain shedding as a function of soluble ectodomain accumulating in the culture medium. While this should primarily reflect proteolytic cleavage of sortilin at the cell surface, a contribution from sortilin shedding initiated in an endosomal compartment cannot be ruled out. In fact, our subcellular fractionations clearly reveal the presence of sortilin ectodomain in intracellular compartments, consistent with previous findings (22). In the cell lysate, we also detected the small sortilin C-terminal fragment that is generated as a result of ectodomain shedding. This C-terminal fragment could potentially also serve as a readout for sortilin ectodomain shedding but intramembrane proteolysis by γ-secretases (58, 59), proteasomal degradation (60) and possible secretion (26, 27) must be considered.

Consistent with previous work by Evans *et al*. (22), showing that ADAM10 is required for sortilin cleavage, we found that sortilin ectodomain shedding is largely dependent on ADAM10 activity. While Evans *et al*. show that sortilin cleavage is independent of ADAM17, our studies demonstrate that sortilin ectodomain shedding is inhibited by TAPI-0, thus implicating involvement of ADAM17. This apparent discrepancy may be due to differential expression of the metalloproteinases in the cell culture systems used. However, because TAPI-0 is also an inhibitor of matrix metalloproteinases, and hence not specific for ADAM17, we cannot rule out a contribution from other proteinases. Inhibition of ADAM10 by GI254023X and ADAM10 knockout abolished the ability of NSG1 to induce sortilin ectodomain shedding. This suggests that NSG1-induced sortilin ectodomain shedding predominantly occurs *via* an ADAM10-dependent mechanism that is likely to be shared with the mechanism responsible for constitutive sortilin shedding. We speculate that NSG1 promotes sortilin ectodomain shedding by facilitating the access of ADAM10 to sortilin's juxtamembrane stalk region (22), possibly mediated by a conformational change in sortilin and/or sequestration of sortilin in specialized lipid microdomains (61). However, further studies are needed to determine the precise molecular mechanism underlying NSG1 induction of sortilin ectodomain shedding as well as the differential effects of NSG1 and NSG2. In addition, it will be interesting to test whether NSG1-mediated proteolysis affects other members of the Vps10p domain receptor family.

In summary, we have identified a novel mechanism for induction of sortilin ectodomain shedding that is regulated by the neuron-specific protein NSG1. Ectodomain shedding can rapidly reduce the level of sortilin cell surface expression and at the same time convert the membrane-associated receptor into a soluble molecule. While the reduction in sortilin cell surface abundance leads to a decrease in sortilin-mediated internalization of ligands, such as PGRN shown in this study, the ectodomain can also affect sortilin-mediated signaling by sequestration and possible neutralization of sortilin ligands and coreceptors. However, it is also conceivable that the sortilin ectodomain can serve to protect ligands from degradation and facilitate transport of ligands over long distances. Inhibition of sortilin-mediated PGRN uptake has been suggested as a possible therapeutic strategy for upregulating PGRN levels in patients with FTD. This has been attempted through the development of mAbs against sortilin. Targeting NSG1 regulation of sortilin ectodomain shedding could represent an alternative therapeutic approach to boost extracellular PGRN levels while retaining the intracellular sorting function of sortilin.

## Experimental procedures

### Antibodies and reagents

Custom primers were purchased from Merck. The In-Fusion HD cloning kit was purchased from Takara Bio. GI254023X and TAPI-0 were purchased from Tocris. Rabbit anti-sortilin (ANT-009) was purchased from Alomone Labs, while goat anti-sortilin (AF2934) was obtained from R&D Systems, both of which recognize the extracellular N-terminal domain. Rabbit anti-sortilin (ab16640), recognizing the intracellular C-terminal domain, was purchased from Abcam. Mouse anti-NSG1 (sc-390654) was purchased from Santa Cruz Biotechnology, goat anti-NSG1 (PA5-37939) from Thermo Fisher Scientific, rabbit anti-NSG1 (LS-B14123) from LS Bio, and rabbit anti-NSG2 (ab189513) from Abcam. Rabbit anti-TfR (#13113), rabbit anti-ERGIC-53 (#13974), mouse anti-Rab7a (#95746), and rabbit control IgG (#2729S) were purchased from Cell Signaling. Rabbit anti-Calreticulin (ab92516), rabbit anti-Vti1b (ab184170), rabbit anti-ADAM10 (ab124695), and rabbit anti-Tubulin (ab4074) were from Abcam. Rabbit anti-Granulin (HPA008763) was from Sigma. Mouse anti-HA (A01244) was purchased from GenScript, while mouse anti-V5 (MCA1360GA) was from Bio-Rad. Chicken anti-MAP2 (822501) was from BioLegend. Rabbit anti-β-actin (926–42210), mouse anti-β-actin (926–42212), and IRDye-conjugated secondary antibodies were purchased from LI-COR Biosciences. The specificity of antibodies used for quantitative evaluations were validated using cell lysate from mock- and construct-specific transfected HEK293MSR cells. Organelle marker antibodies were considered validated if they produced a single band at the expected molecular weight. The specificity of the sortilin ectodomain was further validated using C- and N-terminal directed antibodies (Fig. S5).

### Expression constructs

Human sortilin was obtained from the laboratory of Olav Michael Andersen (Aarhus University) and inserted into pcDNA3.1. Human PGRN in pcDNA3.1(+) was purchased from GenScript. Using seamless cloning a HA epitope tag was added to the 5′ end after the signal peptide sequence to generate an N-terminal HA-tagged PGRN construct (HA-PGRN). Human NSG1 was subcloned from the pPR3 yeast two-hybrid library vector into pcDNA3 and human NSG2 was purchased in pcDNA3.1(+) from GenScript.

### Yeast two-hybrid screen

The DUALhunter system (Dualsystems Biotech AG), which is based on the split-ubiquitin system, was used to identify proteins that interact with the C-terminal cytoplasmic domain of sortilin. Basepairs encompassing residues 779 to 831 of human sortilin were amplified from pcDNA3-sortilin and subcloned into the Sfi I sites of pDHB1, resulting in a fusion protein consisting of a small membrane anchor (the yeast ER protein Ost4), the C-terminal tail of sortilin, the C-terminal half of ubiquitin, and a transcription factor (LexA-VP16) (Fig. S1). The pDHB1-sortilin bait construct was transformed into the yeast strain NMY51. Correct expression of the fusion protein and absence of self-activation was confirmed according to the supplier’s instructions (DUALhunter starter kit user manual). For the yeast-two hybrid screen, a human brain cDNA library (Dualsystems Biotech AG) cloned in pPR3-N (cDNAs fused at the N terminus to the N-terminal half of ubiquitin) was transformed into the NMY51-pDHB1-sortilin strain. Transformants that were able to grow on high stringency selection plates (SD/-Leu/-Trp/-His/-Ade + 5 mM 3-aminotriazole) were tested for β-galactosidase activity. To eliminate the need for plasmid purification, we used direct colony PCR (Phire Plant Direct PCR Master Mix, Thermo Fisher Scientific) to amplify the library cDNAs from positive colonies. PCR products were purified (Purelink Pro 96 PCR purification kit, Thermo Fisher Scientific) and analyzed by automated DNA sequencing. To reveal the identities of the positive clones, the sequences were compared against the National Center for Biotechnology Information database using the BLAST search program. Prey DNA from selected colonies were purified and retransformed into the NMY51-pDHB1-sortilin strain or NMY51 containing the empty pDHB1 vector and grown on selection plates with increasing concentrations of (SD/-Leu/-Trp/-His/-Ade + 1mM-10 mM 3-aminotriazole).

### Generation of ADAM10 CRISPR/Cas9 KO HEK293T cells

The guide RNAs were designed to target exon2 of the human ADAM10 gene (5′-CGTCTAGATTTCCATGCCCA-3′). The NTC sequence was: 5′-TCCGGAGCTTCTCCAGTCAA-3′. The guides for CRISPR/Cas9 were cloned into the lentiCRISPRV2 puro vector (Addgene, cat. no. 52961) according to the lentiCRISPRv2 and lenti Guide oligo cloning protocol (available on Addgene lentiCRISPRv2 website). HEK293T cells were seeded at a 6-well plate. The next day, cells were transfected with 1 μg of the corresponding plasmid using Lipofectamine 2000 reagent according to the manufacturer’s instructions in a ratio of 1 μg DNA:2.5 μl lipofectamine. After 48 h, the medium was changed to a growth medium supplemented with 2 μg/ml of puromycin to select transfected cells. The antibiotic was withdrawn after three days. Cells were maintained for 1 week at their usual growth conditions. The knockout of ADAM10 was confirmed by Western Blot (Fig. S6).

### Cell line cultures and transfection

GripTite 293 HEK293 MSR cells (Invitrogen), ADAM10 KO HEK293T cells and NTC HEK293T cells were cultured as a monolayer in Dulbecco's modified Eagle's medium (Sigma) supplemented with 10% fetal bovine serum (FBS) (Sigma), 0.1 mM nonessential amino acids (Gibco), 1% penicillin/streptomycin (Sigma), and 600 μg/ml Geneticin (only HEK293MSR cells) (Apollo Scientific) under standard conditions at 37 °C and 5% CO_2_. Adherent cells were transfected using EcoTransfect (OZ Biosciences) according to the manufacturer’s instructions. For all experiments, the pcDNA3.1 vector was used to normalize total DNA input per transfection. Cell lines were routinely tested for *mycoplasma* using a quantitative PCR-based service provided by Eurofins.

### SDS-PAGE and Western blotting

All procedures were carried out essentially as described in (62). Aliquots of cell lysates were mixed with SDS sample buffer and incubated at 65 °C for 20 min. The samples were separated on 10% Criterion TGX Precast Protein gels (Bio-Rad) with premixed Tris/Glycine/SDS buffer (Bio-Rad) (Fig. 1, *A* and *B*) or NuPAGE 10% Bis-Tris gels (Invitrogen) using the NuPAGE MOPS or MES buffer system (Invitrogen), and then transferred to 0.2 μm nitrocellulose membranes using the Trans-Blot Turbo Transfer System (Bio-Rad). Membranes were blocked in Odyssey Blocking Buffer (LI-COR Biosciences) and probed with primary antibodies overnight at 4 °C (specified under the separate experiments), followed by incubation with the appropriate IRDye conjugated secondary antibodies (LI-COR Biosciences) for 1 h at room temperature. Infrared signals were visualized with the Odyssey CLx infrared imaging system, and bands were quantified using Image Studio (https://www.licor.com/bio/image-studio) Software Version 5.0.

### Coimmunoprecipitation

Forty hours after transfection, HEK293MSR cells were rinsed with ice-cold washing buffer (50 mM Tris–HCl pH 7.4, 150 mM NaCl) and then incubated with ice-cold lysis buffer (50 mM Tris–HCl pH 7.4, 150 mM NaCl, 5 mM CHAPS, 1× complete protease inhibitor cocktail) at 4°C for 45 min. Cell lysates were cleared by centrifugation at 12,000*g* for 10 min to remove detergent-insoluble material. Equal volumes of total protein lysates were incubated with 2 μg rabbit anti-sortilin (ab16640), 2 μg of rabbit anti-NSG1 (LS-B14123), or 2 μg control rabbit IgG under constant rotation at 4 °C for 2 h. Immunocomplexes were captured by incubating with prewashed protein A-agarose beads (Santa Cruz Biotechnology, sc-2001) at 4 °C overnight. Beads were washed twice in lysis buffer, and bound proteins were eluted with SDS sample buffer (125 mM Tris–HCl, pH 6.8, 30% glycerol, 6% SDS, 0.03% bromophenol blue, and 125 mM DTT) by incubating the samples at 50 °C for 20 min.

Coimmunoprecipitations from rat brains were performed as described above using cleared lysates from prefrontal cortex homogenized in ice-cold lysis buffer (50 mM Tris–HCl pH 7.4, 150 mM NaCl, 5 mM CHAPS, 1× complete protease inhibitor cocktail) using a handhold Kontes microtube pellet pestle.

Aliquots of cleared total lysate and immunoprecipitated proteins were separated by SDS-PAGE and subjected to Western blotting. Primary antibodies used were: rabbit anti-sortilin (1:500; Alomone Labs), mouse anti-NSG1 (1:1000; Santa Cruz Biotechnology), and rabbit anti-NSG2 (1:1000; Abcam).

### Subcellular fractionation

Forty hours after transfection HEK293MSR cells were cooled at 4 °C for 30 min and washed three times with ice-cold PBS supplemented with 1 mM EDTA. Cells were gently detached in ice-cold homogenization buffer (PBS supplemented with 15% sucrose, 1 mM EDTA, 1 protease inhibitor cocktail) using a cell scraper and homogenized by passing the cells ten times through a 0.9 mm syringe. The homogenate was centrifuged (1000×*g*, 10 min, 4 °C), and the cleared lysates were then loaded onto the top of a discontinuous sucrose gradient (15%, 25%, 35%, 45%). After centrifugation (90,000*g*, 18 h, 4 °C, Beckman Coulter TLA100.3 fixed angle rotor), 150 μl fractions were collected from top to bottom of the gradient. An aliquot of the cleared lysate (total protein) and aliquots of individual fractions were separated by SDS-PAGE and subjected to Western blotting. Primary antibodies used were as follow: rabbit anti-TfR (1:1000; Cell Signaling), rabbit anti-ERGIC-53 (1:1000; Cell Signaling), mouse anti-Rab7a (1:1000; Cell Signaling), rabbit anti-Calreticulin (1:1000; Abcam), rabbit anti-Vti1b (1:1000; Abcam), rabbit anti-Granulin (1:200; Sigma), rabbit anti-sortilin (1:500; Alomone Labs), mouse anti-NSG1 (1:1000; Santa Cruz Biotechnology), and rabbit anti-NSG2 (1:1000; Abcam).

### Biotinylation

Cell surface biotinylation: 48 h after transfection HEK293MSR cells were washed three times with ice-cold PBS^++^ (PBS supplemented with 1 mM MgCl and 0.1 mM CaCl_2_) and incubated with 1.0 mg/ml Sulfo-NHS-SS-Biotin (Thermo Fisher Scientific) in PBS^++^ on ice for 30 min with continuous gentle agitation. After labeling, cells were washed twice and incubated with quench buffer (PBS^++^ containing 100 mM glycine) on ice for 30 min. Cells were washed three times with PBS^++^ and lysed with 100 mM Tris–HCl, pH 7.4, 150 mM NaCl, 0.1% SDS, 1% Triton X-100, 1× Complete EDTA-free proteinase inhibitors (Roche Applied Science). The lysate was cleared by centrifugation (12,000*g*, 5 min, 4 °C), and the supernatant was incubated with NeutrAvidin UltraLink Resin (Thermo Fisher Scientific) for 1 h at room temperature. Beads were washed three times in lysis buffer and biotin-labeled proteins were eluted in SDS sample buffer at 50 °C for 20 min. Aliquots of cleared lysates (total protein) and aliquots of biotinylated proteins were separated by SDS-PAGE and subjected to Western blotting. Primary antibodies used were as follow: rabbit anti-sortilin (1:500; Alomone Labs), mouse anti-β-actin (1:2000; LI-COR Biosciences), mouse anti-NSG1 (1:1000; Santa Cruz Biotechnology), and rabbit anti-NSG2 (1:1000; Abcam).

Membrane insertion assay: To measure the rate at which sortilin is inserted into the plasma membrane, cells were washed and labeled with biotin at 4 °C as described above and washed three times with prewarmed PBS^++^ and incubated with PBS^++^ containing 1 mg/ml Sulfo-NHS-SS-Biotin for 0, 5, 10, 15, 20 and 25 min at 37 °C. Cells were quenched and biotin-labeled proteins were isolated and analyzed as described above.

Internalization assay: To determine the internalization rate of cell surface sortilin, cells were labeled with Sulfo-NHS-SS-Biotin and quenched as described above. Cells were washed with prewarmed PBS^++^ and incubated in PBS^++^ for 0, 5, 10, 15, 20 and 25 min at 37 °C to allow endocytosis to proceed. At the end of each time point, cells were washed three times with ice-cold PBS^++^ to arrest endocytosis and treated with the membrane-impermeant reducing agent MESNa (Sigma) twice for 10 min each at 4 °C to remove surface exposed biotin (100 mM MESNa in 50 mM Tris–HCl, 100 mM NaCl, 1 mM EDTA, 0.2% bovine serum albumin, pH 8.6). Cells were washed three times with PBS^++^ and biotin-labeled proteins were isolated and analyzed as described above.

### Progranulin uptake assay

Forty hours after transfection HEK293MSR cells were incubated with conditioned media containing HA-PGRN at 37 °C, 5% CO_2_ for 1 h or 3 h. Cells were washed with ice-cold washing buffer (50 mM Tris–HCl pH 7.4, 150 mM NaCl) and incubated with ice-cold lysis buffer (50 mM Tris–HCl pH 7.4, 150 mM NaCl, 5 mM CHAPS, 1× complete protease inhibitor cocktail) for 45 min at 4 °C. Cell lysates were cleared by centrifugation at 12,000*g* for 10 min, and the supernatants were processed for Western blotting as described above. Primary antibodies used were mouse anti-HA (1:500; GenScript) and rabbit anti-β-actin (1:2000; LI-COR Biosciences).

### Shedding assay

Five hours after transfection, the medium was replaced with one-third volume of fresh medium for concentrating purposes. Forty eight hours after transfection, medium was collected and centrifuged at 6000 rpm for 10 min at 4 °C. Cells were incubated with lysis buffer (50 mM Tris–HCl pH 7.4, 150 mM NaCl, 5 mM CHAPS, 1× complete protease inhibitor cocktail) for 45 min at 4 °C and cleared by centrifugation at 12,000*g* for 10 min at 4 °C. Cleared medium and cell lysates were mixed with SDS sample buffer and sonicated for 3 s and subjected to SDS-PAGE and Western blotting as described above. For treatment with inhibitors, cells grown in fully supplemented Dulbecco's modified Eagle's medium were treated with GI254023X (10 μm and 15 μM), TAPI-0 (15 μM and 25 μM), or 0.1% dimethylsulfoxide for 43 h. Primary antibodies used were: rabbit anti-sortilin (1:500; Alomone Labs), rabbit anti-sortilin (1:500, Abcam), mouse anti-β-actin (1:2000; LI-COR Biosciences), mouse anti-NSG1 (1:1000; Santa Cruz Biotechnology), and rabbit anti-NSG2 (1:1000; Abcam).

### Primary mouse hippocampal cultures

All experimental procedures using vertebrate animals at the University of New Mexico were approved by the Institutional Animal Care and Use Committee. Hippocampal cultures were produced using previously established methods (63). Briefly, brains from WT P0-P1 C57BL/6 pups (The Jackson Laboratory) were isolated, and the hippocampus was dissected in ice-cold Hanks’ balanced salt solution (Sigma) supplemented with 20% FBS and NaHCO_3_ (4.2 mM), Hepes (1 mM; Sigma); pH 7.4. Dissected hippocampi were digested for 10 min with 0.25% Trypsin (Thermo Fisher Scientific), then washed and dissociated using fire polished Pasteur pipettes of decreasing diameter in ice-cold Hanks’ balanced salt solution containing DNase (1500 U; Sigma). The cells were pelleted, resuspended in plating media, and plated at a density of 4 to 5 × 10^5^ cells/12-mm coverslips (Electron Microscopy Sciences) coated with poly-Ornithine (0.1 mg/ml; Sigma, Cat. #4638) and laminin (5 μg/ml; Thermo Fisher Scientific). Cells were allowed to adhere for 15 min before addition of 0.5 ml of plating media containing Neurobasal supplemented with 1× B27, 2 mM Glutamax, 0.5 mg/ml Pen/Strep, and 5% FBS (all from Thermo Fisher Scientific) for the first 24 h. Half of the media was removed and replaced with serum-free media after 24 h. Half of the media was removed and replaced after 48 h with serum-free media supplemented with 4 μM cytosine 1-β-d-arabinofuranoside (Ara-C; Sigma). Neurons were fed by replacing half the volume of spent media with fresh media without serum or Ara-C every week thereafter. For surface labeling of NSG1-2, neurons were transfected using Lipofectamine 3000 (Thermo Fisher Scientific) according to the manufacturer’s specifications, with plasmids expressing human NSG1 or NSG2 linked to the V5 epitope tag on the C terminus.

### Immunocytochemistry

Immunolabeling of fixed primary neurons was performed essentially as described previously (64). Briefly, after fixation (4% paraformaldehyde/4% sucrose), permeabilization (0.2% Triton X-100), and blocking (10% donkey serum in PBS), cells were incubated overnight in primary antibody in 5% donkey serum at 4 °C. Primary antibodies consisted of rabbit anti-NSG2 (1:500; Abcam), goat anti-sortilin (1:500, R&D Systems), goat anti-NSG1 (1:400; Thermo Fisher Scientific), rabbit anti-sortilin (1:1000; Abcam), chicken anti-MAP2 (1:5000; BioLegend). Following primary antibody incubation, cells were washed thrice with PBS and incubated for 1 h with secondary antibody in 5% donkey serum. Conjugated secondary antibodies used were as follow: DyLight 488, 550 and 647 (1:1000; Thermo Fisher Scientific), donkey anti-guinea pig CF555 and goat anti-chicken CF647 (both at 1:500; Sigma). Cells were washed with PBS and then in some cases treated with 4′,6-diamidino-2-phenylindole (1:10,000 in PBS; Thermo Fisher Scientific), followed by three washes with PBS, and mounted on superfrost slides on Fluoromount-G as an antiquenching reagent (Southern Biotech). For surface labeling, primary neurons were allowed to express transgenes for 7 to 8 days and were then treated as above. However, prior to permeabilization, we performed an additional blocking step (10% donkey serum for 30 min), followed by an overnight incubation with goat anti-sortilin (1:1000; R&D Systems) and mouse anti-V5 (1:3000; Bio-Rad) at 4 °C. The cells were then washed three times with PBS, permeabilized with 0.2% Triton X-100 (5 min), and labeled for MAP2 using the methods above for primary and secondary antibody incubations.

### Confocal imaging and analysis

Confocal z-stacks were acquired on the Zeiss LSM800 airyscan confocal microscope using the 63×/1.40NA Oil objective. Sequential frame acquisition was set to acquire an average of ten planes per stack at 16 bit and a minimum of 1024 × 1024 resolution. Channel gain settings were optimally adjusted to minimize saturation of puncta and were maintained across experimental groups. Unmodified images were utilized for all analyses, and linear scaling was applied on images only for presentation purposes using Zen Blue v3.2. Colocalization analysis was performed using the colocalization plugin for ImageJ ComDet (v0.3.4, https://github.com/ekatrukha/ComDet) as previously described (63). Briefly, individual images (single planes) from confocal z-stacks were selected to include those with the greatest number of puncta visible along dendritic arbors. RGB tagged image file format files were color separated, converted to 8 bit, and background subtracted using the sliding paraboloid method in Image J with a rolling ball radius of 50 pixels. For particle detection, regions of interest were then drawn along individual MAP2^+^ dendrites (4–6 per image), with an average width of 5 μm to capture puncta within target arbors and minimize analysis of nontarget puncta. Average particle size was set to six pixels, but larger particles were included and segmented based on average particle size. Intensity thresholds were set manually for each image but were typically 10 to 12 SDs above background. For colocalization the maximum distance between centroids was set to three pixels. Automated detection results were verified manually by at least three individuals not directly involved with the research. For correlation analysis, the same background-subtracted 8 bit tagged image file format files were analyzed using the JACoP (v.2.1.4) plugin for ImageJ, where thresholds were applied manually to images similar to particle analyses.

### Data analysis

All data are shown as mean ± SD. Technical replicates are defined as individual transfections in separate culture wells with the same constructs on the same day. Biological replicates are defined as independent experiments separated in time (typically weeks or months). The mean values from technical replicates were treated as a single measurement in the data analysis of independent biological replicates. Data were analyzed with GraphPad Prism 9.3. Two groups were compared using unpaired two-tailed Student’s *t* test unless otherwise stated. Three or more groups were analyzed with one-way ANOVA, followed by Dunnett’s multiple comparison test or with one-sample *t* test when comparing means of independent experiments expressed as percent of control. *p* < 0.05 was considered statistically significant.

## Data availability

All the data described are contained within the manuscript.

## Supporting information

This article contains supporting information.

## Conflict of interest

The authors declare that they have no conflicts of interest with the contents of this article.
