# Supplementary material for: Neuron-specific gene NSG1 binds to and positively regulates sortilin ectodomain shedding via a metalloproteinase-dependent mechanism
Source: J Biol Chem. 2023 Nov 8;299(12):105446. doi: 10.1016/j.jbc.2023.105446 (PMC10704435; doi:10.1016/j.jbc.2023.105446)
Supplement: Supporting figures [file mmc1.docx]

Supporting Information Figures for

**Neuron-specific gene NSG1 binds to and positively regulates sortilin ectodomain shedding via a metalloproteinase-dependent mechanism**

Malene Overby, Antonio Serrano-Rodriguez, Somayeh Dadras, Ann Kathrine Christiansen, Gözde Ozcelik, Stefan F Lichtenthaler, Jason Porter Weick, Heidi Kaastrup Müller

**Corresponding author**: Heidi Kaastrup Müller, Translational Neuropsychiatry Unit, Department of Clinical Medicine, Aarhus University, Palle Juul-Jensens Boulevard 99, 8200 Aarhus N, Denmark, E-mail: [heidi.muller@clin.au.dk](mailto:heidi.muller@clin.au.dk)

**List of material:**

Supporting Figure 1 (Figure S1)

Supporting Figure 2 (Figure S2)

Supporting Figure 3 (Figure S3)

Supporting Figure 4 (Figure S4)

Supporting Figure 5 (Figure S5)

Supporting Figure 6 (Figure S6)


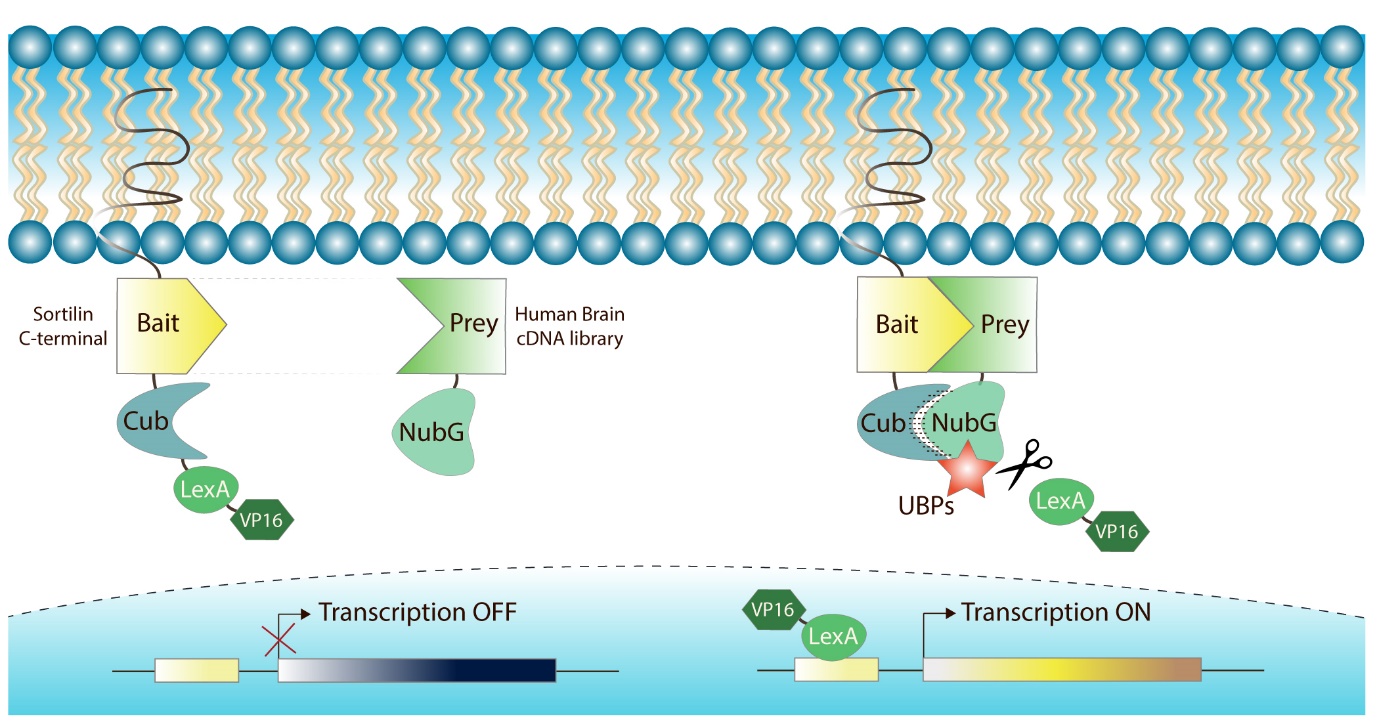


**Figure S1** **- Schematic illustration of the yeast two-hybrid principle**

Schematic illustration of the yeast two-hybrid principle. The C-terminal domain of human sortilin (residue 779-831), corresponding to the bait is fused at its N-terminus to the small membrane protein Ost4p, which anchors the bait at the membrane and at its C-terminus to a reporter cassette consisting of the C-terminal half of ubiquitin (Cub) followed by a transcription factor (LexA-VP16). Potential interacting proteins (preys) are expressed from a human brain cDNA library as fusions to a modified N-terminal half of ubiquitin (NubG), which has no intrinsic affinity for Cub. Upon interaction between the bait and the prey, NubG and Cub are forced into proximity, which results in the formation of split-ubiquitin. Ubiquitin-specific proteases (UBPs) present inside the yeast cell recognize the spilt-ubiquitin and cleave the complex between Cub and LexA-VP16. The released transcription factor can translocate into the nucleus and activate reporter genes, thus converting a protein interaction to a transcriptional readout.


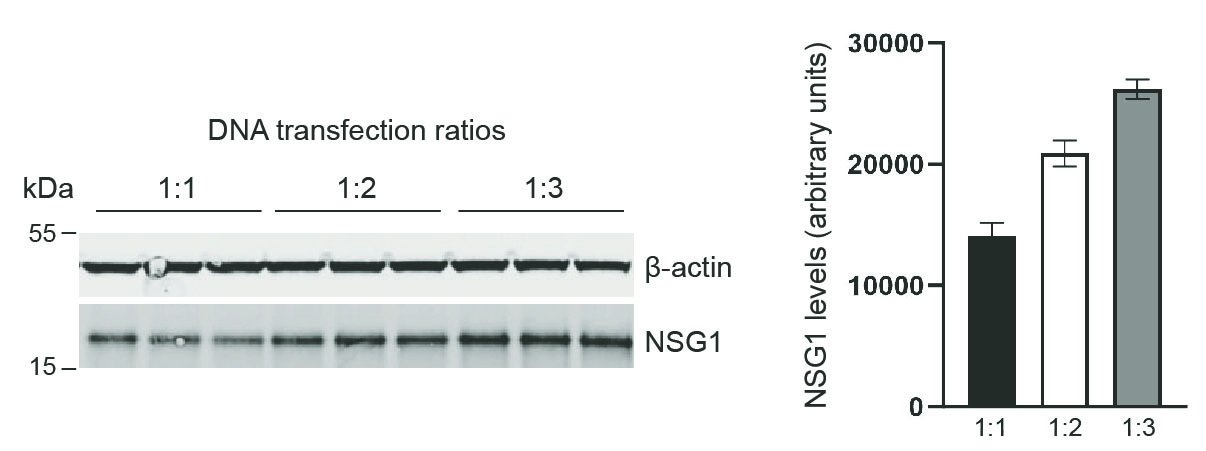


**Figure S2** **–** **Relationship between NSG1 DNA transfection ratios and NSG1 protein expression**

Western blot of cell lysate from HEK293MSR transfected with increasing amounts of NSG1 DNA. NSG1 protein levels increase with increasing DNA transfection ratios. The data are representative of two independent experiments and presented as the mean ± SD of triplicate measurements.


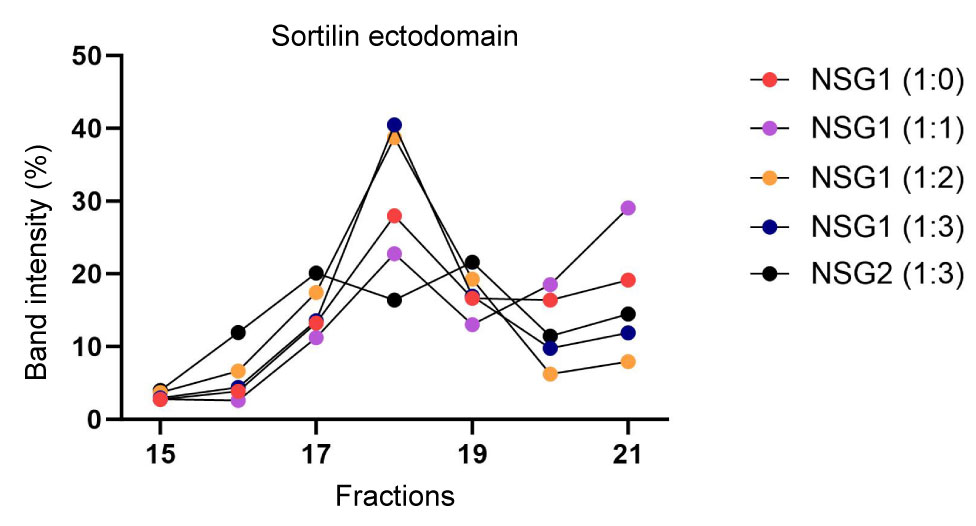


**Figure S3** **–** **Subcellular localization of sortilin ectodomain**

Signal intensities of sortilin ectodomain in fractions 15-21 were quantified and the relative distribution in each fraction is plotted as percentage of total sortilin ectodomain intensity.


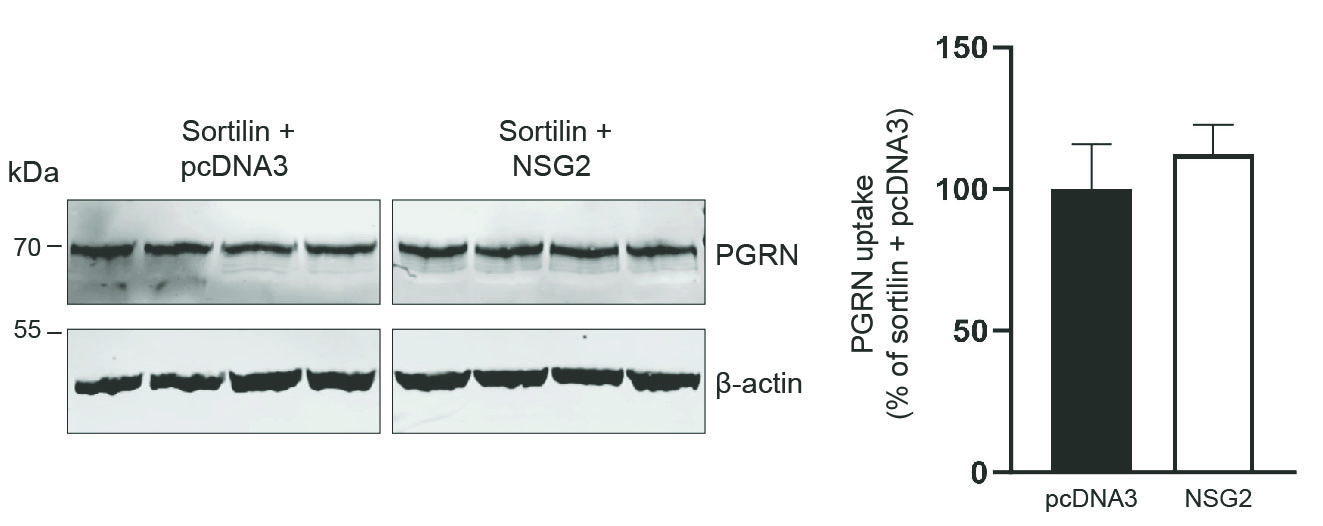
**Figure S4** **–** **NSG2 does not affect sortilin mediated PGRN uptake**

HEK293MSR cells transfected with sortilin and pcDNA3 or sortilin and NSG2 at DNA ratios of 1:3 were incubated with PGRN conditioned media for 3 hours. Internalized PGRN was recovered from whole cell lysates and analyzed by Western blotting. The data are representative of two independent experiments and presented as the mean ± SD of quadruplicates measurements.


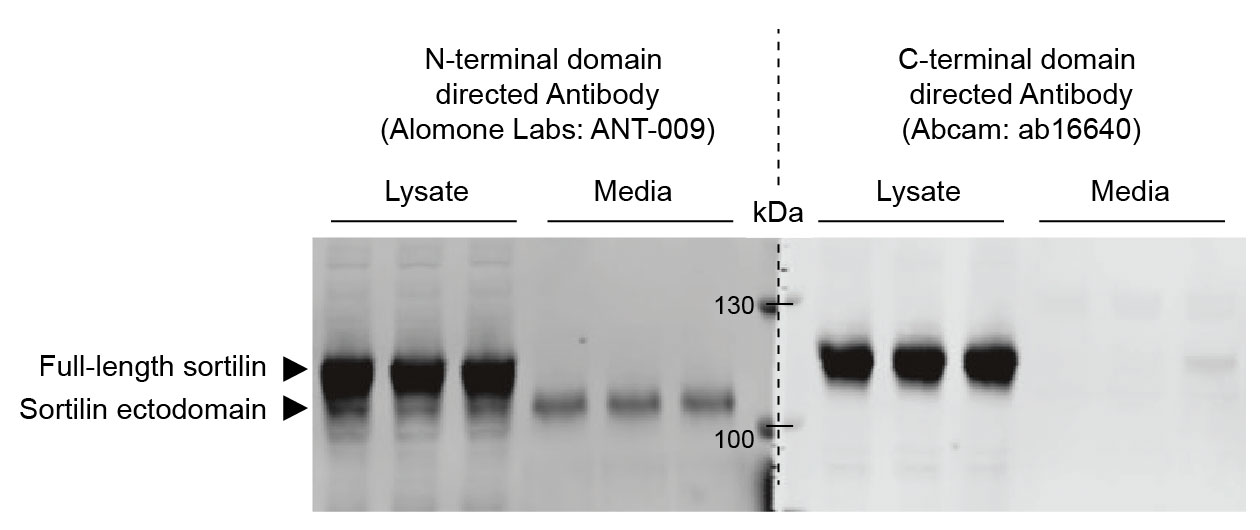


**Figure S5** **-** **Validation of the sortilin ectodomain band**

Western blot of cell lysate and conditioned media from HEK293MSR cells transfected with sortilin. The sortilin ectodomain was recognized in cell lysate and media by the antibody directed against the N-terminal domain of sortilin (Alomone Labs) but not by the antibody directed against the C-terminal domain (Abcam).

**
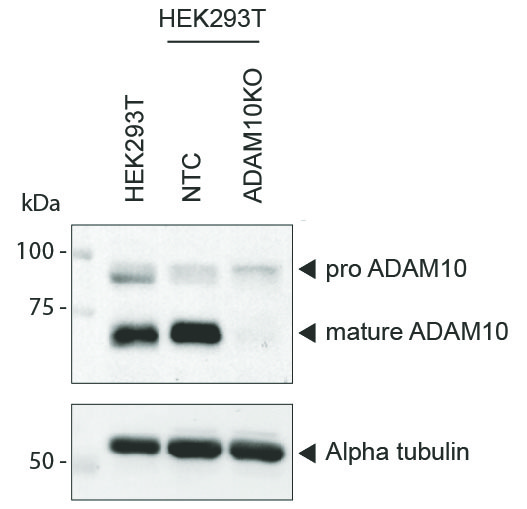
**

**Figure S6** **–** **Western blot validation of CRISPR/Cas9-induced knockout of ADAM10**

Western blot of cell lysate from HEK293T cells, non-targeting control (NTC) cells, and ADAM10 knockout (KO) cells. The figure represents independent cell lysates from each line. Endogenous ADAM10 was detected by an antibody to its C-terminus. Alpha tubulin served as an internal control.
