# Supplementary material for: Neuron-specific gene NSG1 binds to and positively regulates sortilin ectodomain shedding via a metalloproteinase-dependent mechanism
Source: J Biol Chem. 2023 Nov 8;299(12):105446. doi: 10.1016/j.jbc.2023.105446 (PMC10704435; doi:10.1016/j.jbc.2023.105446)
Supplement: Supporting tables [file mmc2.docx]

Supporting Information Table for

**Neuron-specific gene NSG1 binds to and positively regulates sortilin ectodomain shedding via a metalloproteinase-dependent mechanism**

Malene Overby, Antonio Serrano-Rodriguez, Somayeh Dadras, Ann Kathrine Christiansen, Gözde Ozcelik, Stefan F Lichtenthaler, Jason Porter Weick, Heidi Kaastrup Müller

**Corresponding author**: Heidi Kaastrup Müller, Translational Neuropsychiatry Unit, Department of Clinical Medicine, Aarhus University, Palle Juul-Jensens Boulevard 99, 8200 Aarhus N, Denmark, E-mail: [heidi.muller@clin.au.dk](mailto:heidi.muller@clin.au.dk)

**List of material:**

Supporting Table 1 (Table S1)

| **Accession No.** | **Gene** | | **Protein** |
| --- | --- | --- | --- |
| NM_001163.4 | APBA1 | | Amyloid-beta A4 precursor protein-binding family A member 1 |
| NM_001667.4 | ARL2 | | ADP-ribosylation factor-like protein 2 |
| NM_006407.4 | ARL6IP5 | | ADP-ribosylation factor-like GTPase 6 interacting protein 5 |
| NM_001278556.2 | ARPC3 | | Actin related protein 2/3 complex subunit 3 |
| NM_015915.5 | ATL1 | | Atlastin GTPase 1 |
| NM_001689.5 | ATP5G3 | | ATP synthase membrane subunit C3 |
| NM_001256447.2 | BCAP31 | | B-cell receptor-associated protein 31 |
| NM_004052.4 | BNIP3 | | BCL2/adenovirus E1B 19kDa interacting protein 3 |
| NM_001130702.2 | BSCL2 | | Berardinelli-Seip congenital lipodystrophy 2 |
| BC013587.1 | C10orf35 | | Chromosome 10 open reading frame 35 |
| CR533443.1 | C14orf1 | | Chromosome 14 open reading frame 1 |
| NM_001170330.1 | C4orf3 | | Chromosome 4 open reading frame 3 (C4orf3) |
| NM_138493.3 | CCDC167 | | Coiled-coil domain containing 167 |
| NM_001780.6 | CD63 | | CD63 antigen |
| NM_007065.4 | CDC37 | | Hsp90 co-chaperone Cdc37 |
| NM_003885.3 | CDK5R1 | | Cyclin-dependent kinase 5 activator 1 |
| NM_016564.4 | CEND1 | | Cell cycle exit and neuronal differentiation protein 1 |
| NM_006368.5 | CREB3 | | cAMP responsive element binding protein 3 |
| NM_016068.3 | FIS1 | | Mitochondrial fission 1 protein |
| NM_023934.4 | FUNDC2 | | FUN14 domain containing protein 2 |
| CR533519.1 | GHITM | | Growth hormone inducible transmembrane protein |
| NM_001282539.2 | GNB1 | | Guanine nucleotide binding protein G(I)/G(S)/G(T) subunit beta-1 |
| NM_201591.3 | GPM6A | | Glycoprotein M6A |
| NM_001166550.4 | IDS | | Iduronate 2-sulfatase |
| NM_003897.4 | IER3 | | Immediate early response 3 protein |
| NM_206949.3 | IFI27L1 | | Interferon alpha-inducible protein 27-like 1 |
| NM_001134878.3 | KIF9 | | Kinesin-like protein KIF9 |
| NM_001135703.3 | LRP12 | | Low-density lipoprotein receptor-related protein 12 |
| NM_033296.3 | MRFAP1 | | MORF4 family-associated protein 1 |
| NM_007184.4 | NISCH | | Nischarin |
| NM_006392.4 | NOP56 | | Nucleolar protein 56 |
| NM_015922.3 | NSDHL | | Sterol-4-alpha-carboxylate 3-dehydrogenase, decarboxylating |
| NM_001040101.2 /NM_001287763.2 | NSG1 | | Neuron vesicle trafficking-associated protein 1 |
| NM_001134693.2 | OST4 | | Oligosaccharyltransferase complex subunit 4 |
| NM_018930.4 | PCDHB10 | | Protocadherin beta-10 |
| NM_002623.4 | PFDN4 | | Prefoldin subunit 4 |
| NM_015617.3 | PYGO1 | | Pygopus family PHD finder 1 |
| NM_005669.5 | REEP5 | | Receptor accessory protein 5 |
| NM_007033.5 | RER1 | | Retention in endoplasmic reticulum sorting receptor 1 |
| NM_001004333.5 | RNASEK | | Ribonuclease kappa |
| NM_001012456.2 | SEC61G | | Protein transport protein Sec61 subunit gamma |
| NM_021237.5 | SELK | | Selenoprotein K |
| NM_001010897.3 | SERP2 | | Stress-associated endoplasmic reticulum protein 2 |
| NM_000232.5 | SGCB | | Beta-sarcoglycan |
| NM_005827.4 | SLC35B1 | | Solute carrier family 35 member B1 |
| NM_001145432.3 | SMIM20 | | Small integral membrane protein 20 |
| NM_014041.5 | SPCS1 | | Signal peptidase complex subunit 1 |
| NM_005563.4 | STMN1 | | Stathmin 1 |
| NM_001256674.2 | STOML1 | | Stomatin-like protein 1 |
| NM_004853.3 | STX8 | | Syntaxin 8 (STX8) |
| NM_015130.3 | TBC1D9 | | TBC1 domain family member 9 |
| NM_004800.3 | TM9SF2 | | Transmembrane 9 superfamily member 2 |
| NM_016056.4 | TMBIM4 | | Transmembrane BAX inhibitor motif containing 4 |
| NM_003876.3 | TMEM11 | | Transmembrane protein 11 |
| NM_001297551.2 | TMEM128 | | Transmembrane protein 128 |
| NM_032635.4 | TMEM147 | | Transmembrane protein 147 |
| NM_014051.4 | TMEM14A | | Transmembrane protein 14A |
| NM_152464.3 | TMEM199 | | Transmembrane protein 199 |
| NM_001009925.2 | TMEM230 | | Transmembrane protein 230 |
| NM_152766.5 | TMEM256 | | Transmembrane protein 256 (TMEM256) |
| NM_018467.4 | USE1 | | Vesicle transport protein USE1 |
| NM_001134398.2 | VAV2 | | Guanine nucleotide exchange factor |
|  | |  |  |

**Table S1**: List of putative sortilin interaction proteins identified in the yeast two-hybrid screen.
